# Creativity and Generation of Ideas in the Design of Children’s Toys

**DOI:** 10.3390/children10010129

**Published:** 2023-01-08

**Authors:** Bogdan Bucur, Andreea Ban, Sorin Vlase, Arina Modrea

**Affiliations:** 1 Department of the Technology of Information, George Emil Palade University of Medicine, Pharmacy, Science and Technology of Târgu Mureș, 540139 Târgu-Mureș, Romania; 2 Department of Mechanics, Transilvania University of Brașov, 29 Heroes Blvd., 500036 Brașov, Romania

**Keywords:** concept toy-design, creativity, CAD, additive manufacture

## Abstract

Creativity offers new, interesting, and valuable things that can be intangible (ideas, a theory, songs, etc.) or physical objects (a painting, invention, machine). Creativity implies a lot of qualities of the creator such as imagination, creative work, and innovation and it also improves learning and memory. Many of history’s most important discoveries are the results of creative activity. Repetition leads to mastery of a concept through understanding and produces increased self-confidence. Confidence increases the willingness to act on creativity—to explore, discover, and learn. This positive cycle of learning is fueled by the curiosity and enjoyment that comes from discovery and understanding. We are social creatures, so the greatest reward and pleasure comes from the admiration and support received from loved and respected people. Stimulating children’s interest through play also defines solving through exploration regarding the accumulation of new essential information for knowing values and other useful information, by stimulating curiosity and creativity as well as discovering new resources that generate creative ideas, allowing the acquisition of practical skills. All these aspects are oriented and define the premises for the harmonious development of children towards a new existential stage. Thus, taking these aspects into account will have future effects on self-confidence, work strategies, school results, as well as the desire to study and the ability to store and organize accumulated information. The approach of the case study presents through the game, a motivational alternative, staged regarding the generation of creative ideas in the development and materialization of the concept. It is well known that during childhood, many things are acquired by children through selective association and depending on the sensory perception of objects, namely preferred colors, functions, and predefined shapes, proportional to the anthropometric dimensions specific to preschool age. The article proposes the creative approach and generation of ideas on the design of children’s toys; namely, a case study is presented: children’s toy set—teacup.

## 1. Introduction

Creativity is a complex competence. It makes it possible to create real or just mental products, leading to progress in the social area [1,2]. Creativity is, in general terms, the process by which new ideas or concepts are generated, or by which the mind associates existing ideas and concepts. In everyday life, creativity is involved in our life [3,4]. The main component of creativity is imagination, but the creation of real value also implies a motivation, the desire to achieve something new, something special. Creativity has been defined as the interpersonal or intrapersonal process whose results are original and meaningful generating high-quality products [5,6]. Playing is a very important part of children’s lives, as it is a source of joy for the little ones, an excellent source of knowledge and enrichment of knowledge, and has the use of meeting the basic needs of children: the need to manifest themselves, the need to move, the need for encouragement and much more. Playing has an important presence in early childhood education and cannot be replaced by anything else. Personality and the first knowledge of preschool children are formed with the help of games, since at this age the little ones play all the time, being inspired by everyday life. The creative approach through children’s play perfectly combines the phase of assimilation of new information in the cognitive development of children [7]. Knowledge is the factor that develops the motivation to learn, thus it is well known that with the development of computer games the industry, creativity, and socialization are limited in the harmonious and balanced development of children’s character and personality. For these reasons, the physical activities developed by children through playing, form and define skills in the formation of children’s creative character.

For an innovative design, there are situations of creative deadlock, due to the lack of original inspiration for a new perspective. Not often, this crisis of inspiration leads to abandoning the creative idea for a limited or definitive period of time until finding an optimal solution to structure the creative idea. In order to stimulate creativity, techniques and methods are defined that orient the idea towards a structured analysis of ideas that outline the creative result [8,9]. Thus, techniques and methods to stimulate creativity are identified, respectively, the purpose of the approach is to find the perspective of finalizing a positive result, which guides a creative and efficient process associated with the innovative concept [10,11].

### Techniques and Methods for Stimulating Creativity

- The Brainstorming method identifies opportunities to combine ideas so that none is considered an inappropriate idea. The method starts from the premise that each proposal can lead someone else to think of an even better option, so that each contribution is received with respect and openness. The generation of ideas can be conducted using SWOT analyses, and they can be approached in reverse (instead of solving problems, the subjects think about what could cause problems, from a different perspective), thus they try to answer questions such as: who, what, where, when, why and how, as well as encouraging the contribution of all participants.
- The Delphi method presents a creative way to predict the future, whether it is the impact of technology on the coming years, how science will develop, or how society will receive certain changes, the method uses lists of well-thought-out questions to facilitate access to new points of view. In this technique, the participants do not interact, but each has access to the other’s points of view only after they have all completed and answered the questions.
- The Philips method presents a synoptic approach to the creative idea. This method basically plans a brain sprint for the participants involved, who will not tend to waste time with dead moments but will work intensely, trying to bring the best ideas to the minute allotted to them. The time is short enough so that everyone feels motivated, and the number of participants optimal so that it does not seem crowded, or a group without any idea.

The approach of these methods presented through play allows the socialization of children at different ages, at the same time it develops creativity, and the generation of ideas for a rigorous analysis of the proposed concept [12,13]. Obviously, by going through the transitional stages, also through play, the practical skills are fixed and with the advancement in age of the youngest children, in time they will succeed in associating ideas, and creative solutions intended for new projects that require such a creative, innovative structure [14,15]. Stimulating children’s interest through play also defines solving through exploration regarding the accumulation of new essential information for knowing values and other useful information, by stimulating curiosity, creativity as well as discovering new resources that generate creative ideas, allowing the acquisition of practical skills [16,17]. All these aspects orient and define the premises for the harmonious development of children towards a new existential stage [18]. Thus, taking into account these aspects will have future effects on self-confidence, work strategies, school results, as well as the desire to study and the ability to store and organize the information received.

According to the definition [19], a **game** is a recreational activity in which one or more players are involved, being defined by a goal that players try to achieve and a set of rules that determine what players can do. Creative games designed for children have a positive impact on the development of children and their abilities. Through games, the little ones manage to learn about the world and life, and at the same time, they begin to acquire the ability to understand complex problems and themes. Another very important aspect of the game is that it is a very good tool through which children of all ages can develop their fine motor skills. There are a lot of games that stimulate and develop the fine motor skills of little ones. For example, games that include sorting activities or activities that require hand-eye coordination, such as colored drawings. Many recent studies have shown that the development of fine motor skills in children is directly related to the development of thinking, imagination, and communication skills. The source of inspiration for various physical activities, carried out by children, is identified from everyday life [20,21].

Among the first games of childhood, we can identify those related to the environment in which the family is found: the kitchen is considered a source of generating constructive ideas for children, cooking, gardening, and watering flowers show a new interest in the selective ability to perceive the shapes and utility of objects that serve the respective activities and which, in the first phase, are assimilated by copying by the children. The perception of subliminal information also develops a logical, natural connection regarding thinking, motor skills, and the association of ideas, all acquired through play and which later materialize cognitively.

In other words, among the benefits of games we can emphasize:

- a unique and safe way of communication, through games we can talk to children in such a way they can better understand us and they can express their feelings and thoughts, thereby improving the parent–child relationship;
- the child will be able to communicate with other children or adults and therefore develop their communication skills;
- a game is the most beautiful and enjoyable way to learn;
- a game helps children to interact with other children and adults and therefore learn how to create connections with new people;
- a game encourages children to be active and to strengthen and develop their physical condition and creativity.

The game encourages critical and creative thinking. The little ones thus learn to explore different solutions and creative ideas that can be applied to different given problems; the involvement of children in various sports activities has decreased dramatically in recent years, especially owing to the more convenient alternative of **computer games**, **Xbox**, and **Playstation**.

Children no longer ask their parents to buy them a bike, but an Xbox or a computer game that they can use in order to play with other children. The effects are both negative and positive. First, children may become obese from a lack of sports activity, have the wrong diet or become addicted to a certain game [22]. Positive effects include improved dexterity, attention, and imagination. Playing with others, children have beneficial effects on socialization and communication skills through interaction with other children, [23]. In contrast, a virtual game fails to satisfy this need of children and leads to poor communication and a fear of interacting with others. By default, virtual games designed and developed on different platforms of digital technology (computer, tablet, mobile phone, etc.), can lead to apathy, isolation, and a very high addiction. Apathy can affect school performance as well as the long-term personal goals a child has, both among boys and girls (although to a lesser extent) there is a lower interest in sports and a higher interest in virtual games. Although virtual games have positive effects, the disadvantages seem to be more numerous if there is no effective parental control and self-control of children [24,25]. Taking into account the above-mentioned matters, the topic of the article recommends a new approach to team play, which stimulates creativity, curiosity, and abilities of children who can learn to create their own toys, benefiting from the contribution of adults (parents) and new technologies accessible on the market and developed so far, namely 3D prints. Obviously stimulating scientific and technological progress is supposed to be developed with the advent of these skills that are formed through play, through the contribution of each individual to the creative development of such an activity [26]. This paper presents a synthetic study on design, ergonomics, and technological transfer regarding the manufacture of children’s toys. The case study concerns the definition of a tea set for children.

## 2. Materials and Methods

The theme proposes, through the multidisciplinary approach from simple to complex, the assimilation and development of new creative skills, stimulation of curiosity, and motivation of children to give a new direction to team play, combining creativity with technology aiming at harmonious cognitive-functional development of children.

The case study refers to a teacup set composed of 6 reference pieces. The basic idea of the study started from serving tea in the family, transposed into an interactive game between different age groups for children, from the youngest to adults. This aspect involves going through the following stages: hand sketching of the personalized concept, designing and 3D modeling of the components related to the anthropometric dimensions of preschool children, as well as the physical realization through additive technological processes (3D print), and in the second phase the assembly of the components and ready for playing for preparing and serving tea from the new personalized set.

**Figure 1:**
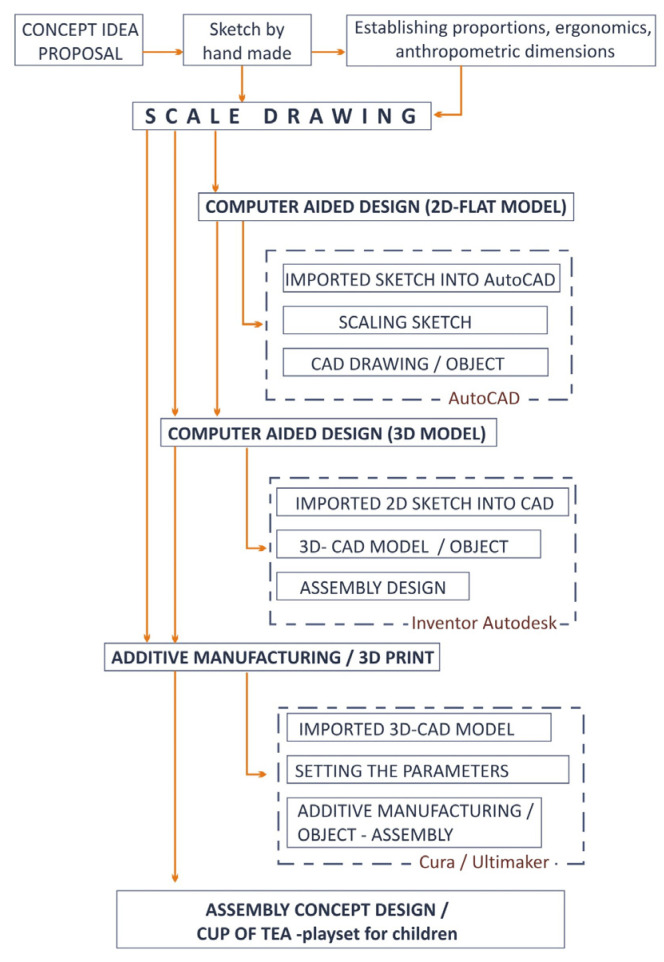
Systemic analysis block scheme.

By the semantic approach to the working methodology [27,28], a gradual transition is made from the idea through the graphic transposition of the sketch by hand to the representation and scale modeling of the sketched objects with the help of programs and digitized technology, and finally, it is proposed to manufacture the entire set in physical format. In Figure 1, a block diagram of the systemic analysis of the methodology is proposed, defining at the same time the working steps, analyzed in the proposed case study.

### 2.1. Drawing Sketch by Hand

The hand-drawn sketch transposes the graphic elements that implicitly define the creative idea of the toy tea set for children and that subsequently allows the project to be produced, while analyzing the proportion, aesthetics, and ergonomics applied to the recommended concept.

Following the synoptic analysis of the sketch, the assembly is identified in an axial section and a top view, to which position 5 and 6 were removed, namely the overall dimensions for each component are defined.

**Figure 2:**
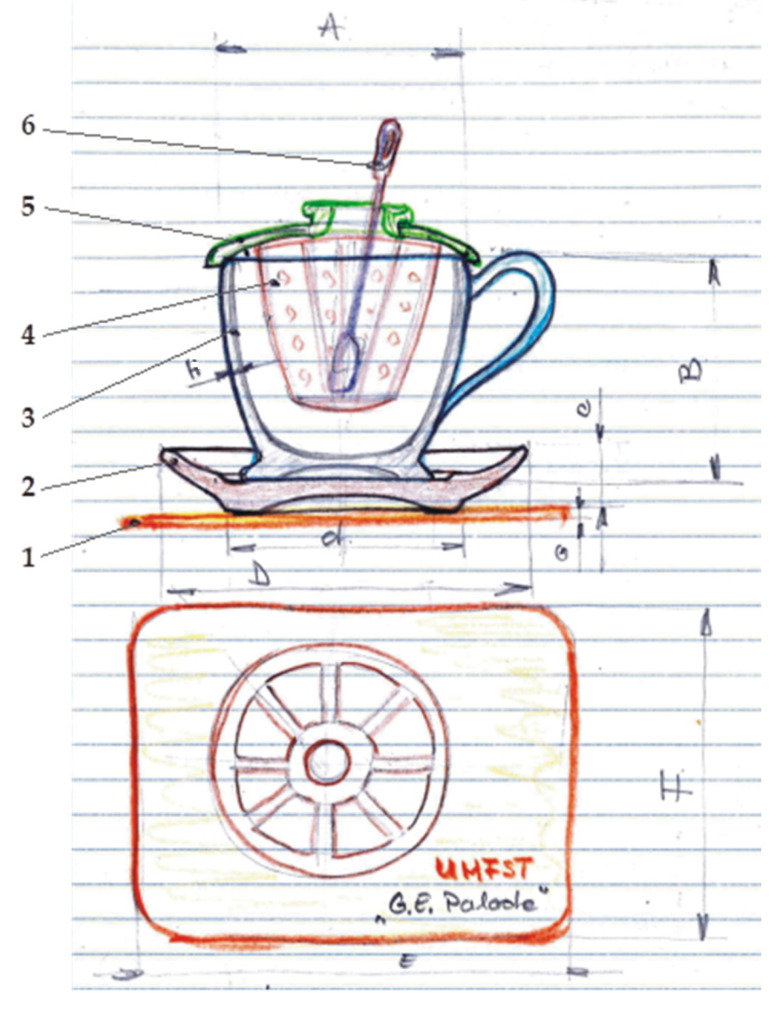
Sketch-proposal concept: set toy for children—teacup: (1) promo biscuit holder, (2) saucer, (3) teacup, (4) separator filter, (5) lid, (6) stirring spatula, A, B, C, D, E, G, c, d, h-geometric dimension.

Figure 2 identifies the hand sketch, which defines the line and the overall geometric shape based on which the project is structured. The geometric curves are freely represented in order to configure the semantics of the ensemble in a well-defined aesthetic and harmony for the intended purpose: children’s toy set, teacup.

The description and composition of the assembly defines 6 pieces, namely: position (1) identifies a rectangular-shaped “promo biscuit” holder, position (2) the saucer, position (3) the teacup represents the basic defining element of the assembly, position (4) presents the separator filter holder through which the infusion of aromas of the plant is made in the quantity of water in the cup, position (5) represents the lid of the assembly with the primary function of closing the assembly and the secondary function of allowing the stirring spatula (6) to be inserted and the contents of the separating filter to be stirred.

### 2.2. Ergonomics and Proportion

The overall dimensions, marked in capital letters on the hand-drawn sketch, subsequently identify the synthetic analysis of the dimensional proportions for each component, as well as the ergonomics of the assembly correlated with the technical anthropometry specific to the anthropometric dimensions of the children (beneficiaries of the play set suggested for development).

Technical anthropometry aims to meet the requirement for children to be able to grasp objects, i.e., that their dimensions are integrated into the comfort dimensions of the objects, while giving them a dynamic operability, and to ensure optimal dimensional adaptation [29,30] through the design details applied to the objects (toys).

**Figure 3:**
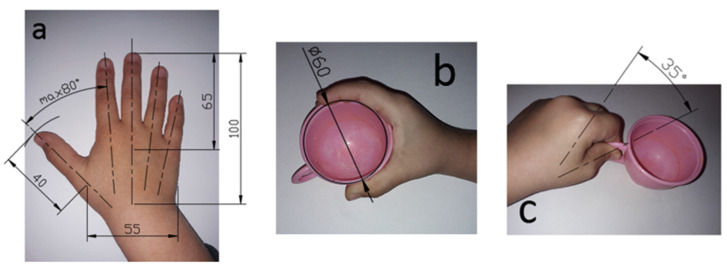
(**a**) Degrees of mobility, (**b**) full-hand grip position, (**c**) finger grip position.

The general principles of applying anthropometry in design work can be systematized and adapted as follows:

- children’s dimensional and functional features (especially hand anthropometry) should be taken into account at an early stage of any project development, as taking them into account at a later stage can be late and often ineffective (Figure 3);
- factors of dimensional variability in both girls and boys must be taken into account;
- all sharp linear or circular edges must be intertwined to eliminate any risk of injury during play.

Anthropometric measures are well defined and there are standard procedures for performing them [26,31]. There are also special tools and equipment for performing measurements.

The ergonomic study provides for the identification of the average dimensions in order to establish the overall dimensions of the parts in the assembly.

The dimensional definition of the parts takes into account the position of the grip with the whole hand, with the fingers, as well as the flexion of the fingers.

The scale setting of the assembly dimensions is defined in a CAD-assisted design environment (AutoCAD, Inventor, Catia, or SolidWorks). On the basis of the hand sketch, the geometric elements are established in the CAD environment [24,32].

### 2.3. 2D/CAD Assisted Design

**Figure 4:**
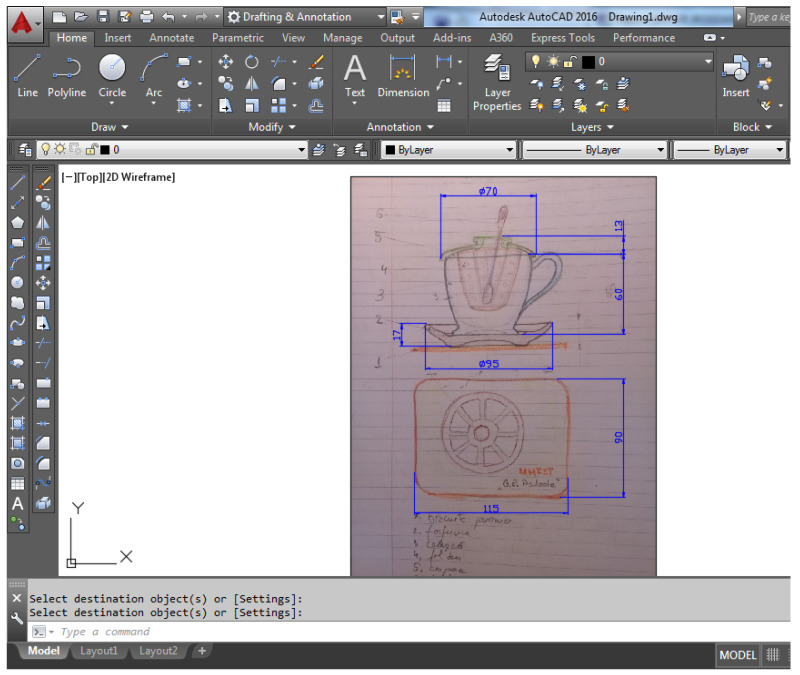
Insert/scale sketch drawing by hand in the AutoCAD environment. (1) promo biscuit holder, (2) saucer, (3) teacup, (4) separator filter, (5) lid, (6) stirring spatula.

At this stage, the dimensional details of the concept sketched by default are established, based on a scan or photocopy of the sketch by hand, (the sketch drawing is executed at an approximate proportion), and the scanned document is imported into the AutoCAD design environment (Figure 4). The work procedure goes through the following stages:

- scanning and importing the sketch by hand into the AutoCAD environment;
- the position A or B is defined dimensionally and by identifying the ratio between the scaled value and the value for dimension A, a unit or subunit ratio, called the scaling factor, is identified, which by application defines the scanned sketch to the considered size, and all dimensions are proportionally reconsidered;
- the geometric elements presented in the section are redesigned on a different layer, so for the squares presented in the section the main dimensions from the whole concept are chosen;
- for each piece identified in the main section, the geometric elements represented at the scale are extracted and then the profile drawings are defined, quoted for each component;
- the end result is all the pieces represented to scale.

**Figure 5:**
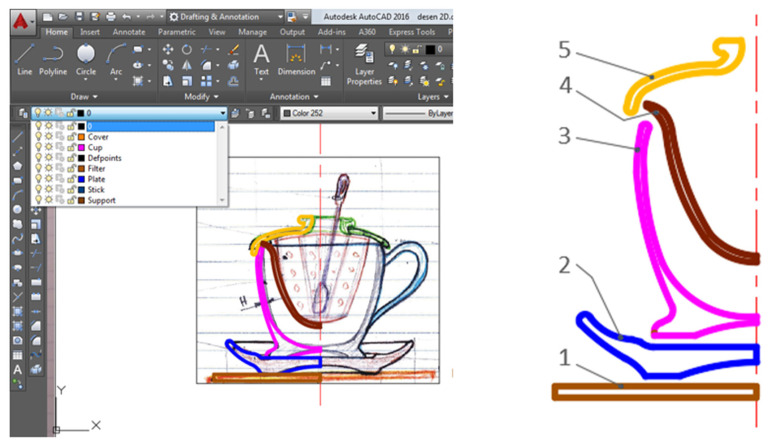
Redesign on different layers of section profiles in the AutoCAD environment: children’s toy set—teacup: (1) promo biscuit holder, (2) saucer, (3) teacup, (4) separator filter, (5) lid.

The 2D design of the part assembly is the preparatory part for the parametric (3D) modeling of the parts in the next step. The scaling factor was defined by setting the dimension A to 70 mm. For a better optimization of the work in the AutoCAD environment, a different layer is defined for each part (layer), thus resulting in 5 geometric profiles, centered along the symmetry axis of the section (Figure 5).

For piece (6), the mixing pestle, the geometrical dimensions are defined separately, respecting the graphic editing proportion, piece (6) is flat, and of simple geometric shape. At this stage of synthesis, all geometric elements are analyzed and finalized, and curvilinear elements, thicknesses, dimensions, and dimensional ratios are corrected.

**Figure 6:**
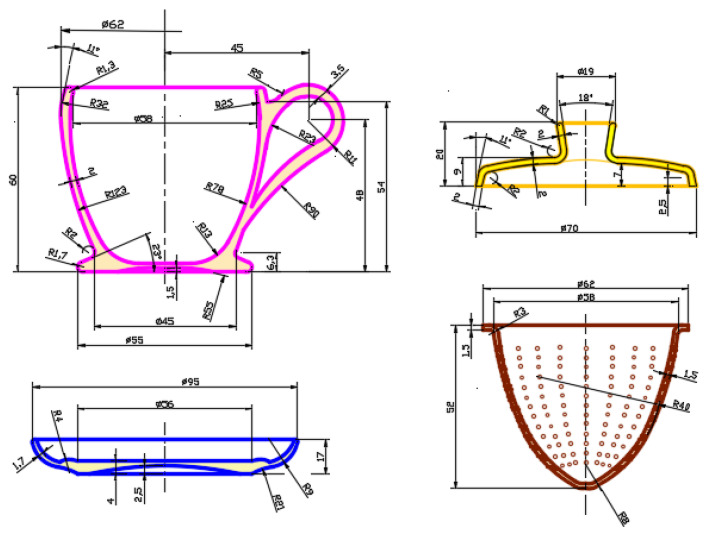
Sizing geometric elements (survey section profiles) in the AutoCAD environment: set toy for children—teacup.

Based on the defined profiles, the assembly execution drawings are drawn up, these profiles are used in the next step for the parametric modeling of each part separately. Figure 6 shows the final geometrical shape of the parts and the dimensioning of the geometrical elements established by the redesign. The symmetry axis is common to all the profiles, so it is the reference element that marks the proportionality of the parts and is imported with the profiles into the 3D parametric modeling environment (Inventor, Catia, SolidWorks, etc.).

### 2.4. Parametric 3D/CAD Modeling

The preparation of the 3D design environment identifies a “project” work file, which allows the automatic saving of all the modeled parts, and in the event that it is desired to diversify/modify the parts it can be carried out optimally, depending on the constraints of the parts.

The Inventor design environment is compatible with the AutoCAD environment, thus simplifying the working procedure for each component part of the sketched assembly. The advantage of using the Inventor environment is that the 3D model can be parameterized if you want to diversify the product in terms of dimensioning, which is not possible in the AutoCAD environment. The import from the AutoCAD environment is performed directly with the “.dwg” extension, preferably the selected profile is of polyline type (block of graphically edited entities).

Based on the profiles resulting from the previous stage, see Figure 5, as well as respecting the dimensions presented in the execution drawings, the 3D models are defined, in the Inventor tool, respecting the geometry and dimensions established as a whole.

**Figure 7:**
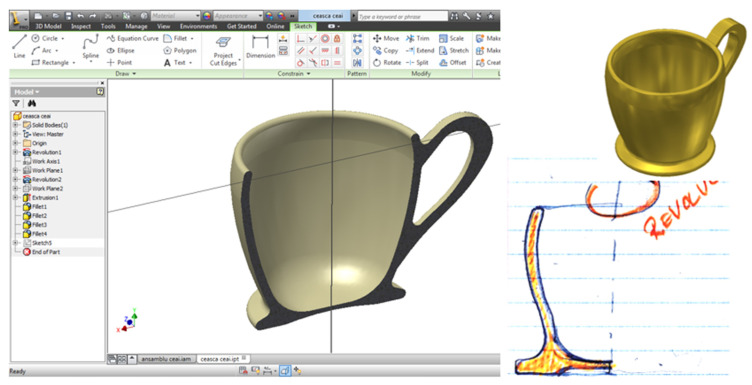
Generating 3D modeling in the Inventor environment.

**Figure 8:**
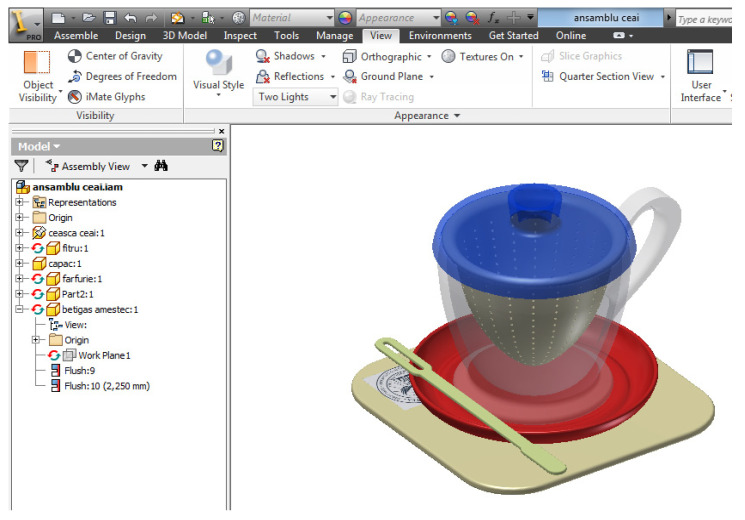
Set toy for children—teacup.

In the Inventor environment, the basic sketch that defines the rotation profile imported from the AutoCAD environment is identified, defining all the dimensional constraints, and in the 3D environment, the translation or rotation procedure of the defined profile is chosen, ultimately resulting in the 3D object. The example in Figure 7 shows the main part of the assembly, the teacup. The work procedure defines three work stages:

1. Importing the generating profile from the AutoCAD environment,
2. Defining the axis of rotation of the generator profile resulting in the body of revolution (teacup),
3. For the handle profile the procedure is identical, importing the geometry from the AutoCAD environment is done in an axial plane of the originally generated 3D body. A new edit file is identified for each part. Once completed, the component parts can be assembled in the Assembly/Inventor environment, inserted and constrained to each other, resulting in the toy set for children—teacup assembly, the result is shown in Figure 8.

**Figure 9:**
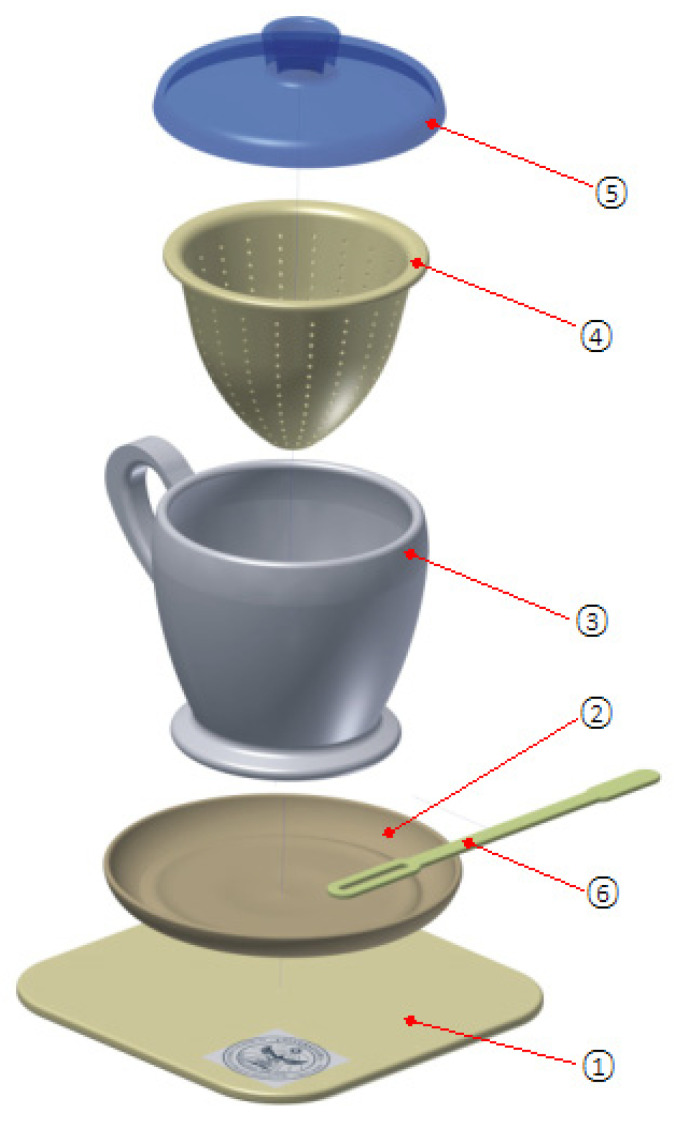
Exploded set: children’s toy set—teacup. (1) promo biscuit holder, (2) saucer, (3) teacup, (4) separator filter, (5) lid, (6) mixing spatula.

**Figure 10:**
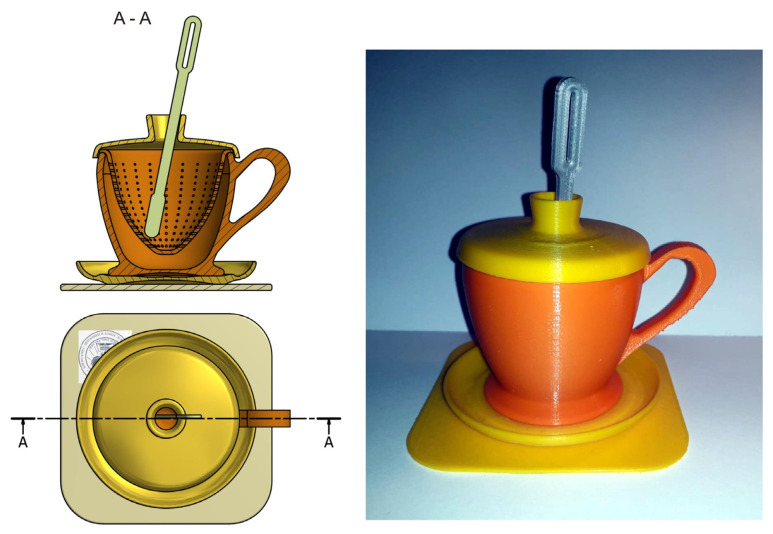
Children’s toy set—teacup. Ensemble and section A-A.

Figure 9 shows the exploded virtual assembly with all six parts represented in isometric projection. In Figure 10, you can see a section through the considered assembly is presented, as well as the actual physical result of the model.

### 2.5. Additive Manufacturing

**Figure 11:**
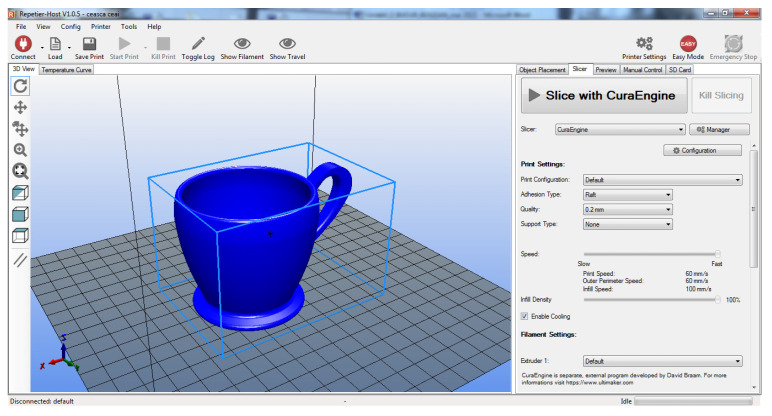
Cura environment interface: setting working parameters.

**Figure 12:**
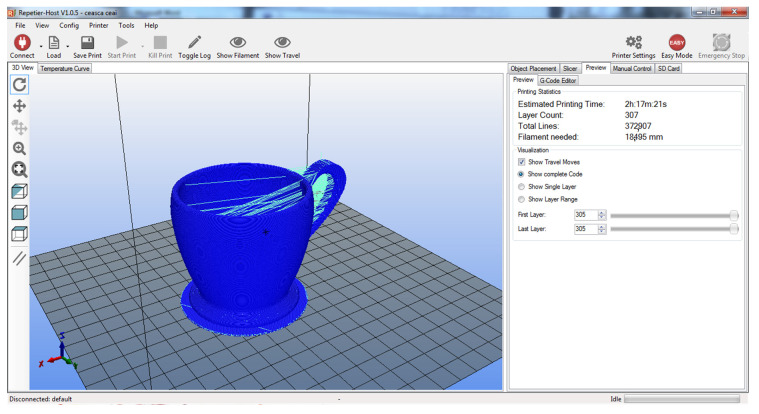
Cura environment interface: final solution.

For the physical fabrication of the entire designed and modeled assembly, additive fusion technology is used for each part considered (Figure 11 and Figure 12).

The 3D models completed in the Inventor environment will be exported with the extension “.STL”, to the CURA environment for setting 3D printer parameters for Ultimaker printers.

For the working procedure, the working parameters are defined in a distinct way, namely:

- orientation of the 3D model ready for printing;
- setting parameters on fabric structure with interlayer fill density as well as extruder travel speed;
- temperature adjustment is another important parameter that must be respected depending on the type of filament used;
- the temperature setting of the printing bed is performed according to the type of filament used;
- choice of filament type: PLA/ABS, different colors preferred;
- code “G”, programming source associated with each 3D model part.

**Figure 13:**
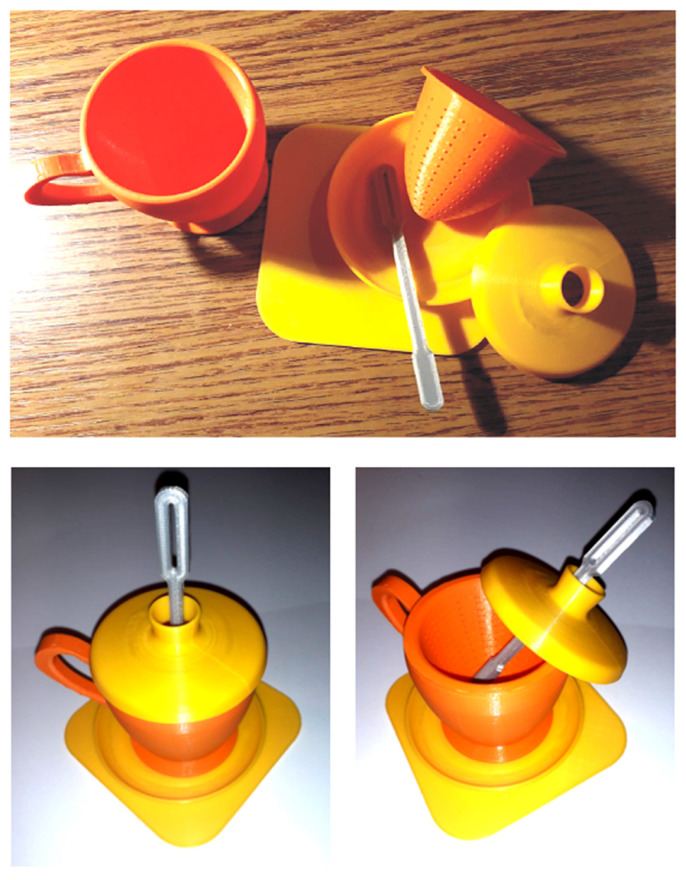
Children’s toy set—teacup (6 component parts).

The orientation of the 3D model on the print bed is chosen so that, as far as possible, the removal of the supports must be removed from the finished piece after 3D printing is performed. This should be avoided as far as possible, depending on the complexity of the part. Figure 13 shows the parts oriented on a self-supporting structure (without support beams).

## 3. Final Results: Assembly Concept Design

The completed project is a creative team learning exercise to diversify children’s playing activities. The materialization of the assembly is done by following step by step the completion of the project: starting from a hand sketch, drawing to scale establishing the proportions in the AutoCAD environment, 3D modeling of each landmark in the Inventor environment, establishing the printing parameters in the Cura environment and finally the production of the physical model of the printed assembly using Ultimaker printers.

Figure 13 shows the physically resulting component parts using Additive Manufacturing technology, PLA filament material, and different colors. The resulting physical model is perfectly functional as long as it is not subjected to thermal shrinkage resulting from high water temperature variations, which can distort the custom shape of the playset components, and custom teacup from the original hand-sketched model. The resulting final assembly represents a complete design and manufacturing exercise applied to a conceptual product dedicated to preschool children’s play and developing the creative side by following and analyzing each stage of teamwork. Based on the model and the systemic analysis method presented in the work, other typologies of more complex structures can be defined that allow the development of imagination, creativity, and spatial vision, at the same time motivating the children’s curiosity and expectations to create the of their own personalized toys. In other words, the exercise represents a self-taught accumulation of information for all team members, which harmoniously complements through play the practical and technical skills at the level of perception and assimilation specific to the age of each member of the team, children and adults alike.

**Figure 14:**
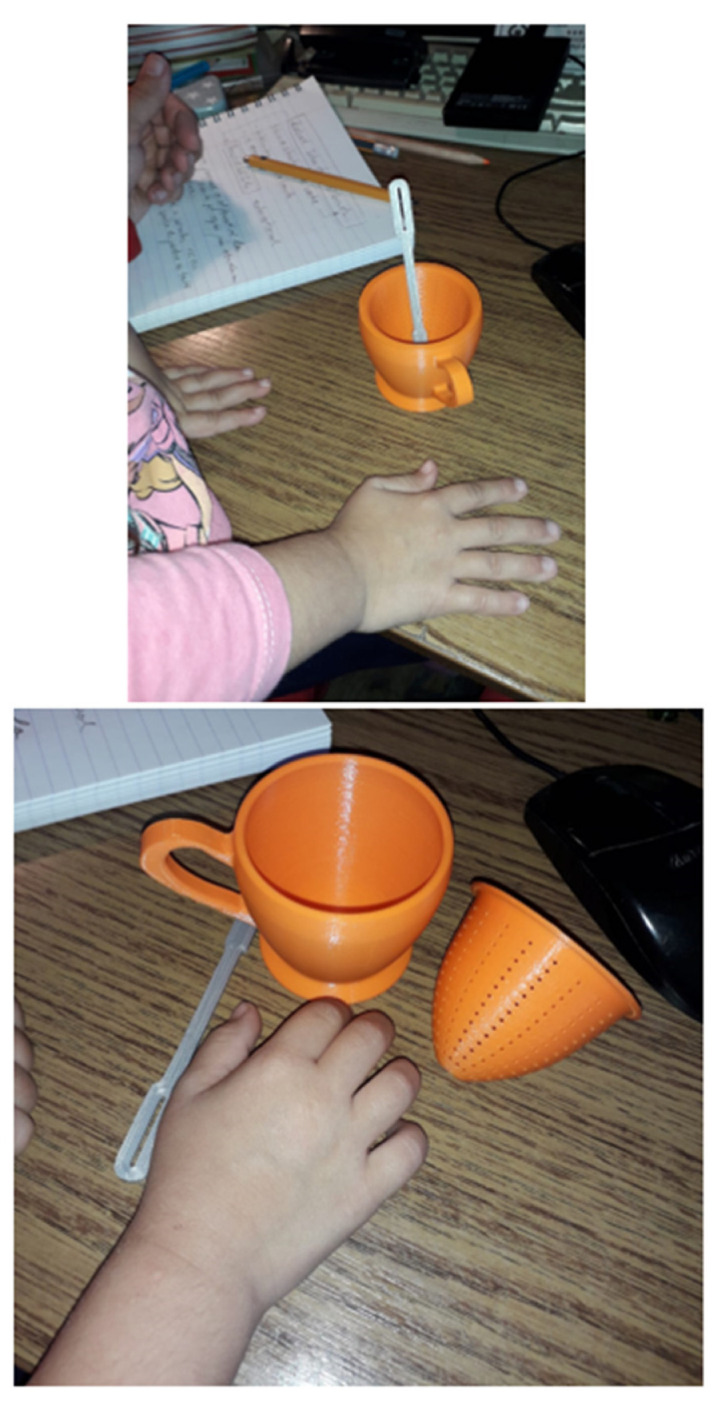
Children’s toy set—teacup.

The definition of colors for the printing filament is done according to other criteria of synthesis and functional analysis preferred by children or by using a combination of specific and appropriate colors for the age of childhood and which creates a pleasant ambience for children during play. Figure 14 shows some snapshots of the children’s play.

## 4. Discussion and Conclusions

The proposed topic has a strong applied character, while developing a new approach to creativity and idea generation aimed at socializing at different pre-school/school ages among children and parents/adults.

Through the step-by-step approach and analysis of the case study, it develops multiple cognitive thinking skills associated with children, who are marked by curiosity and searching for answers.

The multidisciplinary influence implies more elaborate knowledge of the use of CAD programs, as well as the use of what is an additional reason for socializing with adults. Nevertheless, by transferring information children can more easily understand the approach to modern additive design and manufacturing technologies.

The subject matter gradually makes the connection through play, starting from the hand-drawn sketch, it also develops thinking skills through the suggested analysis and synthesis of a hypothetical situation that, in the future, by association, lays the foundation for a creative dynamic character, namely it develops a functional thinking strategy, anchored in social life.

All the screenshots and in-context, graphically edited, scanned images are original, all of which are copyrighted by the authors of the article. The useful time allocated to the additive manufacturing of all the component parts present in the investigated assembly results by adding up the working times for each piece separately, respectively:

Total working time: T_tot_ = T_1_ + T_2_ + T_3_ + T_4_ + T_5_ + T_6_ = 416 [min], about 7 h/set (comprising 6 pieces).

Material: PLA filament, different texture/piece.

## Data Availability

Not applicable.

## References

[1] Kaufman J.C., Glaveanu V. (2022). Positive Creativity in a Negative World. Educ. Sci..

[2] Amabile T.M., Conti R., Coon H., Lazenby J., Herron M. (1996). Assessing the work environment for creativity. Acad. Manag. J..

[3] Sternberg R.J. (2006). The nature of creativity. Creat. Res. J..

[4] Runco M.A., Jaeger G.J., Garrett J. (2012). The Standard Definition of Creativity. Creat. Res. J..

[5] Bogiano A. (1992). Achivement and Motivation, a Social Development Perspective.

[6] Gardner H. (1993). Creating Minds. An Anatomy of Creativity.

[7] Hedges H., Cooper M. (2018). Relational play-based pedagogy: Theorising a core practice in early childhood education. Teach. Teach..

[8] Kaufman J.C., Kapoor H., Patston T., Cropley D.H. (2021). Explaining Standardized Educational Test Scores: The Role of Creativity Above and Beyond GPA and Personality. Psychol. Aesthet. Creat. Arts.

[9] Kaufman J.C., Arrington K.F., Barnett P.J., Holinger M., Liu X.C., Xie L.H. (2022). Creativity Is Our Gig: Focusing on the Positive and Practical. Transl. Issues Psychol. Sci..

[10] Beghetto R.A., Karwowski M., Reiter-Palmon R. (2021). Intellectual Risk Taking: A Moderating Link Between Creative Confidence and Creative Behavior?. Psychol. Aesthetiucs Creat. Arts.

[11] Beghetto R.A., Anderson R.C. (2022). Positive Creativity Is Principled Creativity. Educ. Sci..

[12] Beghetto R.A., Schreiber J.B. (2017). Creativity in Doubt: Toward Understanding What Drives Creativity in Learning. Creativity and Giftedness: Interdisciplinary Perspectives from Mathematics and Beyond.

[13] van der Zanden P.J.A.C., Meijer P.C., Beghetto R.A. (2020). A review study about creativity in adolescence: Where is the social context?. Think. Ski. Creat..

[14] Plucker J. (2020). The Nature of Creativity: Contemporary Psychological Perspectives. Phi Delta Kappan.

[15] Plucker J.A., Peters S.J. (2018). Closing Poverty-Based Excellence Gaps: Conceptual, Measurement, and Educational Issues. Gift. Child Q..

[16] . Bucur B., Ban A., Vlase S., Modrea A. (2022). Aspects Regarding the Innovative Conceptual Design for Children’s Recreational Areas. Children.

[17] Plucker J.A., Jonathan A. (2017). Toward a Science of Creativity: Considerable Progress but Much Work to be Done. J. Creat. Behav..

[18] Sternberg R.J., Lubart T.I. (1996). Investing in creativity. Am. Psychol..

[19] https://ro.wikipedia.org/wiki/Games.

[20] Butterworth G., Harris M. (1994). Principles of Developmental Psychology.

[21] Santash J. (2018). Design creativity: Refined method for novelty assessment. Int. J. Des. Creat. Innov..

[22] https://paverakov.ru/ro/vred-kompyuternyh-igr-na-zdorove-cheloveka-vredny-li-kompyuternye-igry/.

[23] https://www.researchgate.net/publication/255631077_StoryToy_the_Interactive_Storytelling_Toy.

[24] Kroll E. (2001). Innovative Conceptual Design: Theory and Application of Parameter Analysis.

[25] Shwe H. (1999). Smarter Play for Smart Toys: The Benefits of Technology-Enhanced Play.

[26] https://www.scritub.com/profesor-scoala/CREATIVITATEA-SI-MOTIVATIA-FAC35575.php.

[27] https://www.education.govt.nz/assets/Documents/Early-Childhood/Play-ideas/Play-ideas-complete-collection.pdf.

[28] https://lingvo.info/ro/babylon/semantics.

[29] https://en.wikipedia.org/wiki/Human_factors_and_ergonomics.

[30] https://despretot.info/ergonomie-dex-definitie.

[31] dos Santos A.D.P. (2019). Innovation in the Design of Inclusive Toys: Development and Evaluation of a Prototype for Visually Impaired Children. Strateg. Des. Res. J..

[32] Bucur B. (2013). Infografică. Design in AutoCAD.

